# INCREMENTAL VALIDITY OF OPTIMISM AND PURPOSE FOR PREDICTING OLDER ADULTS’ DEPRESSIVE SYMPTOMS

**DOI:** 10.1093/geroni/igz038.1151

**Published:** 2019-11-08

**Authors:** Lauren L Mitchell, Chris Erbes, Paul Arbisi

**Affiliations:** Minneapolis VA Health Care System, Minneapolis, Minnesota, United States

## Abstract

After age 60, depressive symptoms tend to increase slowly over time on average across the population. However, individual trajectories vary, with some increasing more steeply, and others remaining stable. A broad array of psychological constructs have been demonstrated to predict depressive symptoms, including neuroticism, extraversion, optimism, and sense of purpose in life. It is important for psychologists to understand which among these factors are the strongest and most robust predictors. A substantial body of research demonstrates that Big Five personality traits are strongly associated with depressive symptoms (e.g., Hakulinen et al., 2015). Optimism and purpose are also associated with well-being (Carver et al., 2009; Pinquart, 2002), but it is not clear whether such associations could be accounted for by Big Five traits, which are also correlated with optimism and purpose. Using data from the Health and Retirement Study (N = 14,021), we tested the incremental validity of optimism and purpose for predicting older adults’ depressive symptoms, controlling for Big Five traits and demographics. A latent growth curve modeling approach allowed us to examine associations with trajectories of depressive symptoms over six waves (approximately 10 years). Results demonstrated that both optimism and purpose are significantly associated with baseline levels of depressive symptoms, over and above the Big Five. However, only Big Five traits were associated with linear and quadratic slope in depressive symptom trajectories. These findings suggest that optimism and purpose are not redundant with Big Five traits for predicting depressive symptoms, and may be valuable targets for intervention efforts.

